# Clinical Trial Design Principles and Outcomes Definitions for Device-Based Therapies for Hypertension: A Consensus Document From the Hypertension Academic Research Consortium

**DOI:** 10.1161/CIRCULATIONAHA.121.057687

**Published:** 2022-03-15

**Authors:** David E. Kandzari, Felix Mahfoud, Michael A. Weber, Raymond Townsend, Gianfranco Parati, Naomi D.L. Fisher, Melvin D. Lobo, Michael Bloch, Michael Böhm, Andrew S.P. Sharp, Roland E. Schmieder, Michel Azizi, Markus P. Schlaich, Vasilios Papademetriou, Ajay J. Kirtane, Joost Daemen, Atul Pathak, Christian Ukena, Philipp Lurz, Guido Grassi, Martin Myers, Aloke V. Finn, Marie-Claude Morice, Roxana Mehran, Peter Jüni, Gregg W. Stone, Mitchell W. Krucoff, Paul K. Whelton, Konstantinos Tsioufis, Donald E. Cutlip, Ernest Spitzer

**Affiliations:** 1 Piedmont Heart Institute, Atlanta, GA (D.E.K.).; 2 Klinik für Innere Medizin III, Kardiologie, Angiologie und Internistische Intensivmedizin, Universitätsklinikum des Saarlandes, Saarland University, Homburg, Germany (F.M., M. Böhm, C.U.).; 3 Institute for Medical Engineering and Science, Massachusetts Institute of Technology, Cambridge (F.M.).; 4 State University of New York, Downstate Medical College, New York (M.A.W.).; 5 University of Pennsylvania, Perelman School of Medicine, Philadelphia (R.T.).; 6 Department of Medicine and Surgery, University of Milano-Bicocca, Milan, Italy (G.P.).; 7 Istituto Auxologico Italiano Istituto di Ricovero e Cura a Carattere scientifico (IRCCS), Ospedale San Luca, Milan, Italy (G.P.).; 8 Brigham and Women’s Hospital, Boston, MA (N.D.L.F.).; 9 Barts National Institute for Health Research Biomedical Research Centre, William Harvey Research Institute, Queen Mary University of London, United Kingdom (M.D.L.).; 10 University of Nevada/Reno School of Medicine (M. Bloch).; 11 Renown Institute for Heart and Vascular Health, Reno, NV (M. Bloch).; 12 University Hospital of Wales, Cardiff and University of Exeter, United Kingdom (A.S.P.S.).; 13 Department of Nephrology and Hypertension, University Hospital Erlangen, Friedrich Alexander University Erlangen/Nürnberg, Germany (R.E.S.).; 14 University of Paris, Institut national de la santé et de la recherche médicale (INSERM), Centre d'investigation clinique 418, Assistance Publique-Hôpitaux de Paris Hypertension Department and Département médico-universitaire Cardiologie Rein Transplantation Neurovasculaire, Georges Pompidou European Hospital, France (M.A.).; 15 Dobney Hypertension Centre, School of Medicine–Royal Perth Hospital Unit and Research Foundation, University of Western Australia (M.P.S.).; 16 Department of Veterans Affairs and Georgetown University Medical Centers, Washington, DC (V.P.).; 17 Columbia University Irving Medical Center/New York-Presbyterian Hospital, New York‚ NY (A.J.K.).; 18 Cardiovascular Research Foundation, New York (A.J.K., R.M., G.W.S.).; 19 Cardialysis, Rotterdam, The Netherlands (J.D., E.S.).; 20 Thoraxcenter, Department of Cardiology, Erasmus University Medical Center, Rotterdam, The Netherlands (J.D., E.S.).; 21 Department of Cardiovasculaire Medicine, European Society of Hypertension Excellence Center, Princess Grace Hospital, Monaco (A.P.).; 22 Centre for Anthropobiology and Genomics of Toulouse, Toulouse, France (A.P.).; 23 Heart Center Leipzig at University of Leipzig, Germany (P.L.).; 24 Clinica Medica University Milano-Bicocca, Milan, Italy (G.G.).; 25 Division of Cardiology, Sunnybrook Health Sciences Centre (M.M.), University of Toronto, Canada.; 26 Applied Health Research Centre, Li Ka Shing Knowledge Institute of St Michael’s Hospital, Department of Medicine and Institute of Health Policy, Management and Evaluation (P.J.), University of Toronto, Canada.; 27 CVPath Institute, Gaithersburg, MD (A.V.F.).; 28 CERC, Paris, France (M.-C.M.).; 29 Mount Sinai Hospital, New York (R.M., G.W.S.).; 30 Duke Clinical Research Institute, Durham, NC (M.K.).; 31 Departments of Epidemiology and Medicine, Tulane University Health Sciences Center, New Orleans, LA (P.K.W.).; 32 1st Department of Cardiology, National and Kapodistrian University of Athens, Hippocratio Hospital, Greece (K.T.).; 33 Baim Institute for Clinical Research, Boston, MA (D.E.C.).; 34 Beth Israel Deaconess Medical Center, Boston, MA (D.E.C.).

**Keywords:** clinical trials, hypertension, outcomes, renal denervation

## Abstract

The clinical implications of hypertension in addition to a high prevalence of both uncontrolled blood pressure and medication nonadherence promote interest in developing device-based approaches to hypertension treatment. The expansion of device-based therapies and ongoing clinical trials underscores the need for consistency in trial design, conduct, and definitions of clinical study elements to permit trial comparability and data poolability. Standardizing methods of blood pressure assessment, effectiveness measures beyond blood pressure alone, and safety outcomes are paramount. The Hypertension Academic Research Consortium (HARC) document represents an integration of evolving evidence and consensus opinion among leading experts in cardiovascular medicine and hypertension research with regulatory perspectives on clinical trial design and methodology. The HARC document integrates the collective information among device-based therapies for hypertension to better address existing challenges and identify unmet needs for technologies proposed to treat the world’s leading cause of death and disability. Consistent with the Academic Research Consortium charter, this document proposes pragmatic consensus clinical design principles and outcomes definitions for studies aimed at evaluating device-based hypertension therapies.

Despite its recognition as the leading cause of death and disability worldwide, the awareness, treatment, and control of blood pressure (BP) have plateaued if not modestly declined.^1,2^ In the United States alone, for example, more than half of individuals with hypertension—representing >29 million people—are estimated to have BP exceeding professional society and guideline-recommended treatment goals.^3,4^ As the most commonly diagnosed condition and largest contributor to mortality in industrialized countries, hypertension is present in >1 in 3 individuals, and the risk of cardiovascular mortality doubles for every 20 mm Hg and 10 mm Hg increase in the systolic BP (SBP) and diastolic BP (DBP), respectively.^5^ With population growth and aging, the global prevalence of hypertension and associated adverse outcomes are expected to continue to escalate.^6^

Irrespective of baseline hypertension severity, even moderate reductions in BP translate into clinically meaningful reductions in cardiac, renal, and cerebral vascular-related adverse events.^7,8^ Recent large randomized clinical trials have also demonstrated the clinical benefits of more intensive BP reduction,^9,10^ leading to revision of guideline-directed treatment goals to further lower SBP and DBP standards.^11,12^ Countering evidence-based efforts to improve BP with a broad spectrum of pharmaceutical therapies are routine challenges in clinical practice that include patient intolerance of medication-related adverse effects, general nonadherence with prescribed therapy, and physician inertia for treatment of an illness that remains largely silent until onset of clinically irreversible conditions.

## Device-Based Therapies for Hypertension

Over the past decade, the potential utility of device-based hypertension therapies has evolved from enthusiasm to skepticism and then renewed promise. After several observational studies and an unblinded randomized trial^13^ suggesting clinically significant reductions in BP among patients with severe treatment-resistant hypertension,^14–16^ renal denervation (RDN) was positioned as an important new treatment strategy with global public health effect, and early enthusiasm for clinical adoption outpaced the available data. After the lack of demonstrable effectiveness compared with a sham procedure and antihypertensive therapy in the SYMPLICITY HTN-3 trial (Renal Denervation in Patients With Uncontrolled Hypertension),^17^ however, the prospect of RDN’s role in clinical practice diminished and appeared to validate earlier criticisms of trial conduct and, more broadly, the prospects for device-based hypertension therapies.

**Figure 1.**
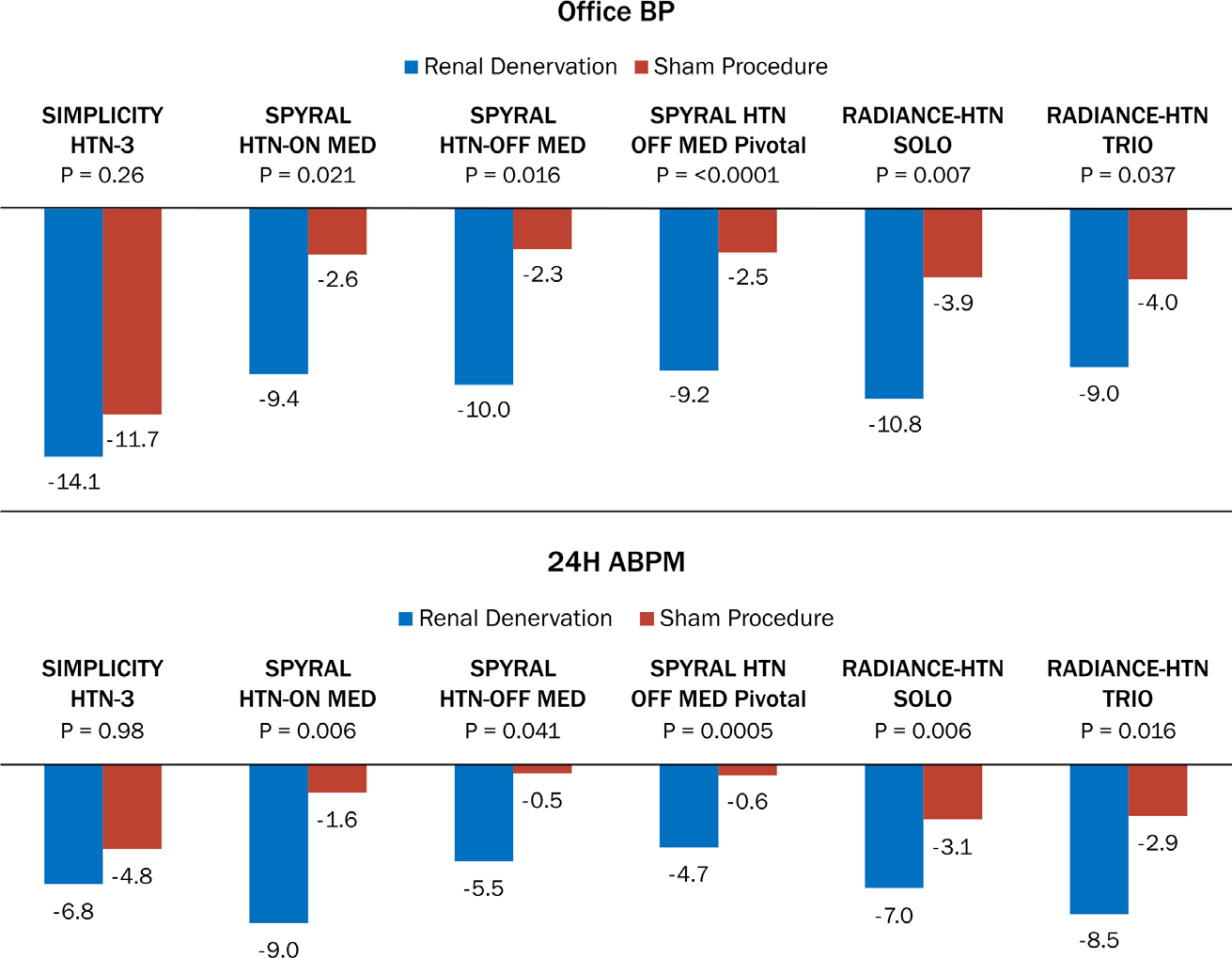
Change in systolic blood pressure (mm Hg) after renal denervation in 6 prospective, randomized, sham-controlled trials. ABPM indicates ambulatory blood pressure measurement; BP, blood pressure; RADIANCE HTN SOLO, Study of the ReCor Medical Paradise System in Clinical Hypertension in Absence of Hypertension Medications; RADIANCE-HTN TRIO, Study of the ReCor Medical Paradise System in Clinical Hypertension in the Presence of a Stabilized, Single Pill, Triple, Fixed Dose Antihypertensive Medication Regimen; SYMPLICITY HTN-3 trial, Renal Denervation in Patients With Uncontrolled Hypertension; SPYRAL HTN-OFF MED, Global Clinical Study of Renal Denervation With the Symplicity Spyral Multi-Electrode Renal Denervation System in Patients With Uncontrolled Hypertension in the Absence of Antihypertensive Medications; SPYRAL HTN-OFF PIVOTAL, Global Clinical Study of Renal Denervation With the Symplicity Spyral Multi-Electrode Renal Denervation System in Patients With Uncontrolled Hypertension in the Absence of Antihypertensive Medications Pivotal; and SPYRAL HTN ON MED, Global Clinical Study of Renal Denervation With the Symplicity Spyral Multi-Electrode Renal Denervation System in Patients With Uncontrolled Hypertension on Standard Medical Therapy. Adapted from references 15, 17–21.

Advances in trial design and conduct to account for confounding variables of procedural technique, medication variability, and selection of both patients and outcomes have substantially informed subsequent studies.^18^ Additional exploratory preclinical and clinical studies continued, and signals of effectiveness with RDN persisted, motivating further investigations. To date, results from 5 sham-controlled, randomized RDN trials have demonstrated significant BP reductions in patients on or off concomitant antihypertensive therapies (Figure 1),^19–23^ supporting a biological proof of principle for this novel treatment. In addition to RDN, device-based therapies for which the effectiveness and safety are being evaluated in randomized controlled trials include those targeting carotid sinus baroreceptors, and pacemaker modulation of atrioventricular intervals.^19–21,24,25^ Table 1 summarizes completed and ongoing clinical trials of these device-based therapies. Given this background, experience with device-based therapies for hypertension warrants a careful approach to clinical evidence generation with a thoughtful consideration of patient population, trial methods, and procedural safety and effectiveness outcomes.

**Table 1.**
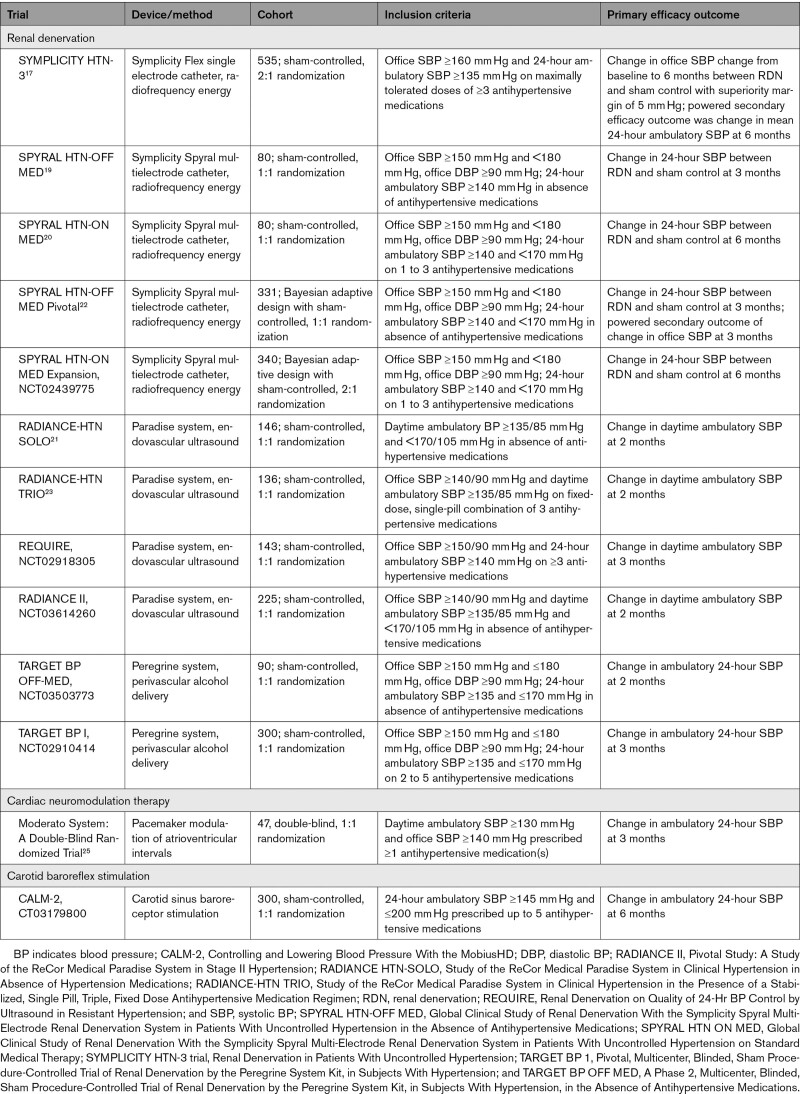
Contemporary Randomized Trials of Device Therapies for Hypertension

## Hypertension Academic Research Consortium

## Rationale and Methods

Consistent, pragmatic definitions that support key clinical trial processes such as independent adjudication of study outcomes and safety oversight provide informative benefit/risk evidence and can promote efficient innovation of safe and effective therapies. The Hypertension Academic Research Consortium (HARC) was initiated to create consensus among experts involved in developing device-based therapies for hypertension following the process defined in the ARC charter.^26^ In February 2020, HARC participants met in in Silver Spring, MD. The meeting was organized by Cardialysis (Rotterdam, The Netherlands) and was attended by experts from Europe, the United States, Canada, and Australia, as well as representatives from the US Food and Drug Administration, a European Notified Body (DEKRA Certification BV, Arnhem, The Netherlands), and observers from the cardiovascular device industry (participant listing in the Supplemental Material). After the meeting, focused writing groups were charged to develop individual sections on outcomes definitions and trial design principles presented in this document.

## Clinical Trial Design Consideratons

### Trial Design, Conduct, and Presence or Absence of Medications

**Figure 2.**
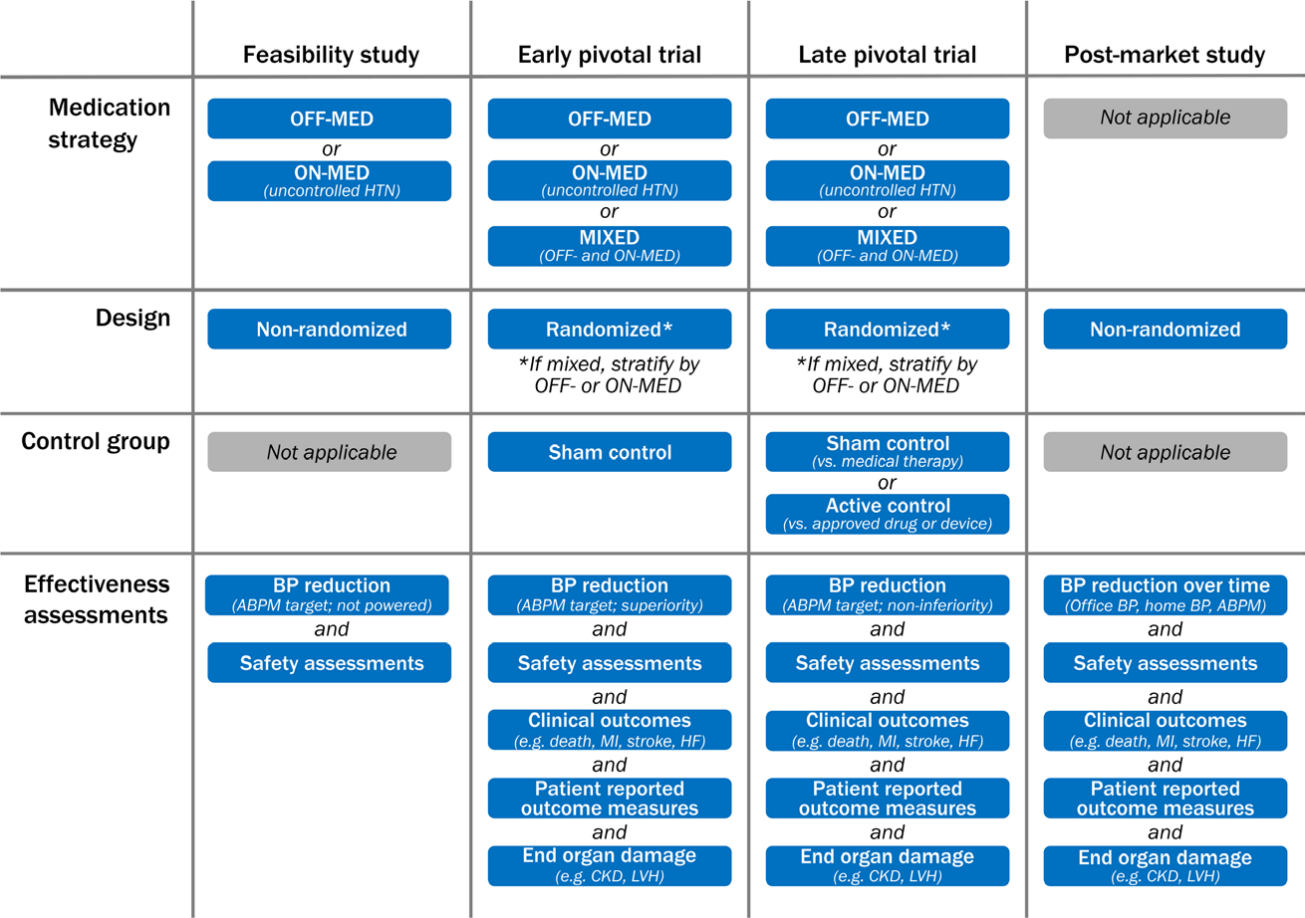
Clinical investigation recommendations for novel devices for hypertension management. ABPM indicates ambulatory blood pressure measurement; BP, blood pressure; CKD, chronic kidney disease; HF, heart failure; HTN, hypertension; LVH, left ventricular hypertrophy; and MI, myocardial infarction.

The design for clinical trials of device-based therapies for hypertension treatment will vary depending on the regulatory phase of strategy. Figure 2 outlines many of the specifics recommended by HARC for the various stages of hypertension device therapy clinical studies.

Initial studies to test proof of concept and feasibility are typically single-arm or open-label randomized trials.^14,24,27^ Notable features of feasibility studies include a target population similar to that planned for pivotal studies and an adequate number of participants to demonstrate some evidence of effectiveness and assessment for major safety concerns, although statistical significance is not required. During feasibility and pilot studies, modifications of the technology or the procedure may be tested with finalization of the device design and procedural protocol before a pivotal trial.

HARC recommends a staged off medications (OFF-MED) then on medications (ON-MED) progression of randomized trials. After a first stage of addressing safety and biological plausibility (OFF-MED), recruitment of an ON-MED population of patients with uncontrolled hypertension is logical to document the magnitude of the antihypertensive effect of the procedure and its durability in the presence of medications. In practice, recruitment of patients with uncontrolled hypertension prescribed a single agent of the same class would be impractical, and thus most ON-MED studies allow patients on 1 to 3 (or more) antihypertensive medications. These designs are prone to large between-patient variability in BP response after the procedure. An alternative, used in the RADIANCE HTN TRIO study (Study of the ReCor Medical Paradise System in Clinical Hypertension in the Presence of a Stabilized, Single Pill, Triple, Fixed Dose Antihypertensive Medication Regimen),^23^ is to switch at enrollment all eligible patients with apparent resistant hypertension prescribed multiple medications to a triple combination single pill to be taken for the remainder of the study. This strategy promotes medication adherence and a more consistently treated cohort of patients with resistant hypertension confirmed by ambulatory BP (ABP).

Early phase studies may be followed by randomized, blinded, sham-controlled trials to compare effectiveness and to assess device safety (OFF-MED and ON-MED). Although a 1:1 allocation of experimental treatment versus control is the most efficient design, a 2:1 (study device versus control) randomization ratio may be considered to enlarge the size of the study cohort (achieving learning curves faster and affording more patients exposed to the device-based therapy to identify adverse safety outcomes), although this will necessarily increase sample size to achieve statistical power to demonstrate effectiveness in BP lowering. Additional safety data can be obtained by “crossing over” control group patients after measurement of the primary effectiveness outcome to receive the device-based therapy or allowing for access to the device-based therapy in either the premarket or the postmarket interval.

The US Food and Drug Administration requires sham-controlled trials whenever feasible and ethical, and pivotal clinical trials for at least the first generation of device-based therapies must therefore include a sham procedure group as the control.^28^ Results from a recent meta-analysis suggested that in most interventional trials, sham procedures are safe, and the use of the sham procedure when applied in a symmetrical way without cointervention after randomization produces a robust estimate of the effect, albeit smaller than in nonsham trials and more likely closer to the true effect.^29^ The most appropriate placebo control for device-based therapies is an invasive sham procedure, mimicking the active treatment closely. For RDN trials, typically a sham involves a renal angiogram but no denervation device advanced into the artery. The use of invasive sham procedures is generally associated with a higher degree of complexity of trial execution, introducing ethical concerns of performing a procedure conferring an immediate risk of adverse events and potential harm without potential benefit to the patient. However, by blinding the patient and all postprocedure assessors, sham-controlled trials reduce confounders and bias, allowing the true treatment effects of any interventional therapy to be discerned. Although nearly all recent hypertension trials of device-based therapies have been sham-controlled,^19–23^ it is important to recognize this 1 design attribute does not always exclude other flaws in trial design or performance.

Once safety and effectiveness in both on and off medication blinded, pivotal trials has been proven, subsequent randomized trials may be open-label with a conventionally treated control group^13^ or include a control group with strict protocol-specified medical therapy.^30^ For trials involving medical therapy, adherence testing using urinary or plasma metabolite assays may be useful to interpretation of results. Among blinded studies, effectiveness of maintaining the blind must be demonstrated through patient questionnaires administered immediately after the procedure and at the time of the primary outcome assessment. For open-label studies, a Prospective Randomized Open-label Blinded End Point (PROBE) design should be used with assessment of BP response by an observer-independent BP measurement such as ABP monitoring (ABPM) and by evaluators blinded to the randomization.^30^

Once RDN technologies are approved and are available in clinical practice, sham or medical therapy controls may no longer be required or feasible. Instead, a shift from device versus medical therapy to investigational device versus approved device controls is anticipated. Once this occurs, HARC advises that it is reasonable to propose an active device control using a noninferiority design. In RDN device versus device trials, however, treatment assignment should remain blinded to the patients, research staff, and subsequent caregivers, if possible. Such noninferiority designs must be rigorous, requiring evidence of effectiveness of the active comparator on the primary outcome of interest with strict noninferiority margins as well as adequate clinical outcome and safety data.

Single-arm studies are generally not considered as proof of safety or effectiveness for a device-based therapy, particularly in the early phases of developing breakthrough technologies such as current RDN methods. More frequently, single-arm studies are performed as postapproval device-based studies to provide broader insight into the effectiveness of a therapy in a less selected patient population and in scenarios common to clinical practice. Well-designed registries can recruit larger volumes of patients than randomized trials, identifying signals of rare but serious adverse events that might provide guidance, especially in the postmarketing phase. They may also allow subgroup hypotheses to be generated for effectiveness.

### Medication Burden and Adherence

The BP response to device-based therapies cannot be reliably quantified without accounting for antihypertensive treatments prescribed in terms of the number of different classes of antihypertensive medications as well as doses, and patient adherence to the prescribed medications in both blinded and unblinded periods of a trial. Accounting for medication changes is particularly challenging after unblinding, because differential behavior of health care providers or patients when knowing treatment allocation may lead to a potential bias, thus obscuring the intervention’s effect per se, especially in the long term.

Medication burden is usually evaluated using simplified dichotomous measures (either on- or off-treatment), or a simple measure of the number of antihypertensive medications (disregarding the dose and the class), not accounting for the dose-dependency of the BP-lowering effect of the medications. Besides these simple measures, medication burden can also be evaluated using the sum of defined daily dose of each individual antihypertensive medication and by using the antihypertensive load index,^31^ which is the sum of the ratio of the current daily dosage divided by the maximum recommended daily dosage for each medication. The maximum daily dosage of each agent as indicated for hypertension is obtained from pharmaceutical databases.^31,32^ Although alternative models may be developed, medication indices should account for both number and dose of prescribed medications.

Nonadherence to medications is recognized as a major factor in reducing BP treatment effectiveness and contributes to perceived “treatment resistance” in patients with hypertension. Indeed, nonadherence is highly prevalent (in up to 50% of patients), especially in patients diagnosed with severe hypertension.^33^ In individuals with perceived treatment-resistant hypertension, 50% of treatment resistance may be explained by nonadherence (“pseudoresistance”).^34^ In addition to adverse clinical events, there are numerous adverse consequences of suboptimal adherence to antihypertensive medications including a confounding effect on BP outcomes in clinical trials of device-based therapy for hypertension. Moreover, the time course of poor adherence can be variable, with fluctuating adherence over time, further confounding measurements of effectiveness.^20^

Reasons for poor adherence to treatment are multifactorial. Treatment-, patient-, provider- and health care system–related factors affect medication nonadherence in patients with hypertension, but predicting nonadherence remains challenging. Several indirect and direct methods have been developed to assess treatment adherence (Table S1). Assessing medication adherence helps guide medical treatment, avoid unnecessary and potentially harmful treatment intensification, decrease the number of medical visits at specialized clinics, and allow implementation of strategies to improve medication adherence. Medication monitoring and informing patients about testing have been shown to improve BP control, and adherence to antihypertensive medications is associated with lower cardiovascular risk.^35^ Inclusion of adherence assessment in clinical trials as described in this section is important for interpreting study outcomes, at least in the early development phase of device-based therapies, to inform the treatment effect.

### Medication Reinstatement Algorithms

Except for patient-related safety requiring deviation from study protocol, baseline medications should remain without change (or absent in OFF-MED trials) for both treatment and control groups until timing of the primary BP outcome measure. There are 2 main reasons for starting, resuming, or enhancing medical therapy after the study’s primary outcome is reached: (1) an ethical need to control BP in the trial’s study participants, and (2) to compare medication requirements between the device-treated and the control groups to learn whether the procedure affects the intensity of medical treatment required to achieve the BP target.

A typical medication algorithm, already used in device-based hypertension trials for patients initially OFF-MED,^19,21,22^ is described in Table S2. The selection of medication classes, and their order of use in this stepwise titration, conforms with widely used current international hypertension guidelines,^11,12^ with further recommendations provided in the Table S2 footnote how to individualize therapy in patients with difficult-to-control hypertension. One shortcoming in trials completed to date^19,30^ is that using the algorithm has been left to the discretion of the clinical investigators and not mandatory. It is recommended that future trials include rigorous protocols for the intensification of medical therapy by the clinical staff or treating physicians, as tolerated, to achieve target BP, while verifying adherence to medications by patients.

## BP Outcomes for Device-Based Therapies for Hypertension Trials

The following section addresses the specific advantages and disadvantages of each method to measure and report BP and potential complementary use of multiple BP assessment strategies.

### Office BP

When properly performed, office BP (OBP) measurement is inexpensive and accurate, and reflects what has been used in landmark clinical trials and routine clinical practice for decades. In early RDN trials, however, OBP results as a primary outcome often demonstrated higher variability than in traditional medical therapy studies, leading to uncertainty about consistency in measurement and method, susceptibility to regression to the mean, and inability to confirm treatment effect. This variability may have been amplified by inconsistent medication regimens and adherence.

The importance of using proper technique cannot be overstated and has been described elsewhere.^36^ Strict adherence to measurement protocols makes choice of “attended” or “unattended” (ie, with or without a health care provider present) readings less important, but the same approach should be consistently applied throughout a clinical trial. Averaging data from >1 visit increases precision and reduces sample size but may also increase trial complexity.^37^ Effects associated with unblinded assessment and white-coat effect should be minimized.

In randomized trials, the mean difference in BP from baseline to specific posttreatment time points (eg, 3 or 6 months) compared between treatment and control groups is a measure of effectiveness. SBP reduction is generally the key clinical target, but DBP is of particular interest in trials that enroll younger patients or use elevated DBP as an entry criterion.

Data from both observational and randomized studies, including meta-analyses of medical therapy trials, demonstrate that decrements in office SBP of 5 and 10 mm Hg are associated with ≈10% and ≈20% reductions in cardiovascular disease events, respectively, and independent of comorbidities such as cardiovascular disease.^7^ As such, a mean difference in OBP of at least 5 to 10 (preferably closer to 10) mm Hg for SBP compared with baseline, or 3 to 5 mm Hg for DBP, may be considered a clinically meaningful reduction. In addition, BP response rates (ie, responder analysis) and time within target range can provide a complementary effectiveness assessment.

### Ambulatory BP

ABPM yields more precise and reproducible BP profiles than conventional OBP assessments.^38^ ABPM is less susceptible to bias and placebo effect and eliminates the white-coat effect.^39^ It estimates short-term BP variability and provides better prognostic value for end-organ damage and cardiovascular events versus OBP.^40^ In addition, the relationship between cardiovascular events and mortality is stronger for ABPM than OBP, in particular when including nocturnal BP.^40–42^

For these reasons, ABPM has been adopted as the preferred BP outcome in major randomized RDN trials. ABPM has proven to be particularly well suited because the BP-lowering effects of RDN have been apparent throughout a 24-hour period.^43^ Although elevated SBP and DBP are associated with increased cardiovascular risk, the association with SBP is more consistent and persists after adjustment for DBP. Therefore, SBP has been preferred in hypertension clinical trials as the primary outcome.^12^ In RDN trials, both ambulatory daytime and 24-hour SBP assessments have been used.

A reduction in mean 24-hour ambulatory SBP of ≥5 mm Hg relative to baseline can be considered a clinically meaningful response to RDN,^28,44,45^ recognizing that a 10-mm Hg reduction in office SBP is associated with a significant reduction in the risk of major cardiovascular events and mortality across varying baseline BPs.^7^ The procedures to measure ABPM have been previously described.^36^ To address issues of adherence and missing data, 24-hour ABPM has been considered valid with a minimum of 20 daytime readings and 7 during sleep.^46,47^ Independent core laboratories are desirable to provide feedback on data quality, maintain uniform process across sites, conserve confidentiality, and reduce potential bias.

### Home BP

Home BP measurement has emerged as an important complement to OBP and ABPM in both clinical practice and research. Self-measured home BP avoids the white-coat response, allows for multiple measurements over time, identifies patients with masked hypertension, and has been shown to correlate better with target organ damage and cardiovascular events than OBP.^36^ However, home BP measurement is highly operator-dependent and is reliant on equipment accuracy; home BP also misses nighttime BP, orthostatic hypotension, and short-term BP variability. Awareness of home BP results may also bias patient behaviors and medication adherence. On the basis of these limitations, home BP measurement can be a secondary outcome for device-based therapy trials, but assessment issues limit its use as a primary outcome.

Optimal home BP measurement methods have been previously detailed.^11,12,36,48^ At each outcome assessment time, it is important to record BP twice daily (morning and evening) for at least 7 consecutive days with the readings from the first day discarded in clinical trials,^46^ and at least 70% of expected readings at each time period should be available (ie, up to 30% missing data are not ideal but currently acceptable). Electronic transfer of readings to an independent core laboratory is encouraged and may enable long-term effectiveness follow-up without requiring participants to attend a study visit.^49^

Evidence from the relatively few observational studies relating cardiovascular risk with home BP measurement suggest that each 10-mm Hg increase in SBP is associated with an ≈15% to 20% increase in adverse events.^50^ Given their similar profiles on determination of cardiovascular risk,^51^ similar thresholds for clinically significant response may be considered for daytime ABP and home BP in device-based therapy trials. Table S3 describes the advantages and disadvantages ABPM and home BP measurements.

### Consensus Summary: Selection of BP Measurement Modalities as Outcomes

Clinically relevant BP reduction considerations proposed by consensus are represented in Table 2. All BP assessments—ambulatory, office, and home—may be considered for trials evaluating device-based hypertension treatment because each modality offers different but complementary information on the BP response (Table S4). As a practical matter, however, all randomized trials of hypertension therapies may use 2 or more of these measures, typically with ABPM specified as the primary effectiveness outcome measure.

**Table 2.**
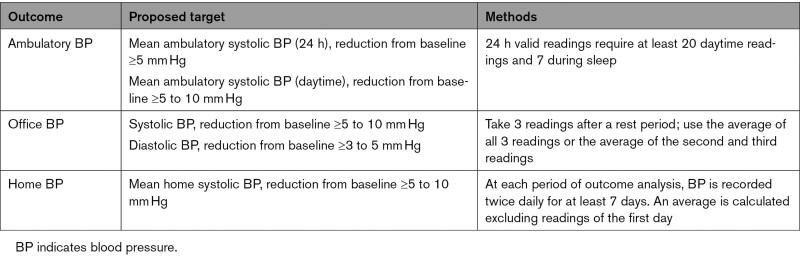
Blood Pressure Outcome Considerations in Device-Based Therapies for Hypertension Trials

## Additional Outcomes Relevant to Device-Based Therapies for Hypertension Trials

### Clinical Outcomes

HARC recommends to ascertain and classify death,^52,53^ neurological events,^54^ myocardial infarction,^55^ bleeding,^56^ access and vascular complications,^56^ acute kidney injury,^56^ and heart failure,^47^ following standardized definitions (Tables S5–S8).^52,53,57^ Although complications occur infrequently with current technologies, procedure-specific criteria are proposed on the basis of existing ARC standards and available in the Supplemental Material. Independent adjudication of clinical events (both safety and effectiveness) is a quality marker in clinical trials, enhances the validity of study results, and is often requested by regulatory authorities.

### Target-Organ Damage

Regression of hypertension-mediated organ damage is associated with improved cardiovascular outcomes.^58^ Several prospective observational studies have suggested that RDN is associated with regression of echocardiographic or cardiac magnetic resonance imaging–based left ventricular mass index,^59,60^ improvement in left ventricular function,^59–61^ decrease in left atrial volume index,^61^ and augmentation index or pulse wave velocity,^59^ irrespective of BP reduction.^62^ These studies, however, are limited because of sample size and lack of independent, blinded core laboratory assessment. Available data, limited by sample size and short-term follow-up, suggest that RDN is safe and effective in patients with chronic kidney disease, although it remains to be established whether RDN leads to improvement in albuminuria or kidney function, or at least attenuates the decline in glomerular filtration rate.^63^

### BP Targets and Variability

Clinical guidelines for hypertension treatment share an OBP target of <130/80 mm Hg (when tolerated and on the basis of risk profiles), with an OBP of <140/90 mm Hg for those >65 years of age per the European guidelines.^11,12,47^ The proportion of patients achieving these targets is an alternative to targeting a specified absolute BP reduction from baseline,^11,44^ considering that relative percentage reduction from baseline after device-based therapies is influenced by initial BP levels and might be less applicable in milder forms of hypertension.^19^

Long-term visit-to-visit variability in BP offers prognostic information in patients with hypertension and can be used as a secondary efficacy outcome. High BP variability has been associated with increased cardiovascular risk,^64^ and device-based therapies have been shown to reduce such variability after correction of baseline BP values, thus offering potential as an effectiveness outcome (Table S9).^65^ A more consistent reduction in BP would expectedly mitigate BP variability and improve time in target range that has been associated with a lower risk of adverse events.^66^

### Heart Rate

Aside from reductions in both sympathetic activity and BP after RDN, a decline in resting heart rate has also been shown in several studies.^67,68^ In the SPYRAL HTN-OFF MED pilot study (Global Clinical Study of Renal Denervation With the Symplicity Spyral Multi-Electrode Renal Denervation System in Patients With Uncontrolled Hypertension in the Absence of Antihypertensive Medications), for example, resting heart rate was reduced after RDN both in the office and during daytime and nighttime ABPM.^68^ From 2 reports, an elevated resting heart rate (above the median 73.5 beats per minute) has also been suggested to predict BP reduction after RDN.^68,69^ Other reports, however, have not demonstrated this association. At present, heart rate reduction after device-based therapy should be considered an exploratory outcome.

### Predictors of Treatment Response

The inability to accurately identify individual therapy responders undermines efforts to identify broadly applicable predictors of response and inform patient selection. To date, post hoc “responder” analyses on the basis of single-point paired comparisons may not appropriately characterize a treatment response given (1) day-to-day BP variability, (2) the lack of consensus about follow-up time point or meaningful reduction in BP, (3) dependency of the change in BP from the pretreatment BP, (4) the suggestion that even lesser declines in BP may not imply absence of a treatment effect, and (5) susceptibility of treatment bias.^70^ Unlike the population mean effect that statistically compensates for BP variability, an individual patient can only be compared against a random time point that could be higher or lower than the patient’s long-term mean BP. In the SPYRAL HTN-OFF and ON MED (Global Clinical Study of Renal Denervation With the Symplicity Spyral Multi-Electrode Renal Denervation System in Patients With Uncontrolled Hypertension on Standard Medical Therapy) trials^19,20^ and the RADIANCE HTN-SOLO^21^ (Study of the ReCor Medical Paradise System in Clinical Hypertension in Absence of Hypertension Medications) trials, for example, approximately half of patients in the sham group at outcome follow-up experienced a BP increase, whereas half experienced a decrease, and few patients had no change. Although often impractical, averaging multiple BP measurements at baseline and follow-up might be required to control for BP variability and for regression to the mean, and specifically identify individual treatment responders.

A broad list of potential predictors of response has been proposed to date but is limited with little if any external validation. No individual predictor, except baseline BP, has been consistently identified across multiple trials. In the SPYRAL HTN-OFF MED Pilot and Pivotal trials, ambulatory heart rate above the median (>73.5 bpm) was predictive of reduction in average daytime SBP, daytime DBP, and office SBP.^69^ Recently, both plasma renin activity and aldosterone levels were significantly reduced after RDN compared with sham control; higher baseline plasma renin activity was associated with a significantly greater reduction in both office and 24-hour SBP.^71^

Last, because no reliable procedural measure exists to confirm effective nerve ablation, the extent to which incomplete denervation contributes to clinical nonresponsiveness is uncertain. HARC endorses incorporation of exploratory substudies as part of larger trials confirming safety and effectiveness.

### Patient-Related Outcomes and Preference

Patient perspectives and preferences are important in assessing the benefits and risks of antihypertensive therapy. In general, patient-reported outcomes, quality of life, and patient preferences have until recently rarely been the main objective of clinical trials in device-based medicine. Patient-reported outcomes instruments rely on patients’ response to questionnaires (eg, quality-of-life questionnaires), and on measurements of patients’ well-being (eg, cognitive function) or outcomes self-assessed through medical diaries/ trackers. To date, the European Quality of Life 5-Dimension 3 Level (EQ-5D-3 L) questionnaires and the short form health survey (SF36) are considered to be reliable and validated measures of quality of life and have been used in hypertension research.^72^ In a recent survey, patient and physician attitudes toward pharmaceutical therapies and RDN for hypertension may be differ relative to severity of hypertension and medication burden.^73^ Specifically, preference for device-based therapy among patients may be highest among those not taking medications and may be independent of BP severity.

HARC encourages incorporating such assessments in device-based hypertension trials and developing health status models specific to this area of study. For patient preference, Discrete Choice Experiments may be useful to quantify patients’ preferences for attributes of interventional and pharmaceutical treatments for hypertension that include both effectiveness and risks of treatment-related adverse events.^74^ For self-reported outcomes, the first step is the selection of an appropriate patient-reported outcome measure (PROM), presenting the patient’s perspective about how an interventional procedure for hypertension will affect their well-being. The selection of a PROM is based on its appropriateness, acceptability to patients, interpretability, validity, and ability to detect changes over time.

For future trials, HARC recommends the following: (1) the validation of PROMs in the field of hypertension that account for the heterogeneity of patients’ perception according to culture or health care systems, (2) systematic inclusion of PROMs in prospective trials, and (3) inclusion of patient’s perspective and preferences at an early stage of clinical trial design.

## Timing of Assessments Related to Late-Term Safety and Effectiveness

### Timing of Outcome Selection

Four key factors may be considered when determining the optimal timing for outcome assessment in device-based hypertension trials: (1) what the study population is (eg, on versus off medications, mild versus severe hypertension); (2) what the primary assessment is (BP versus measures of end-organ damage); (3) when the treatment effect is expected; and (4) what the likelihood is of confounders interfering with the treatment effect. Timing of outcome recommendations is provided in Table 3.

**Table 3.**
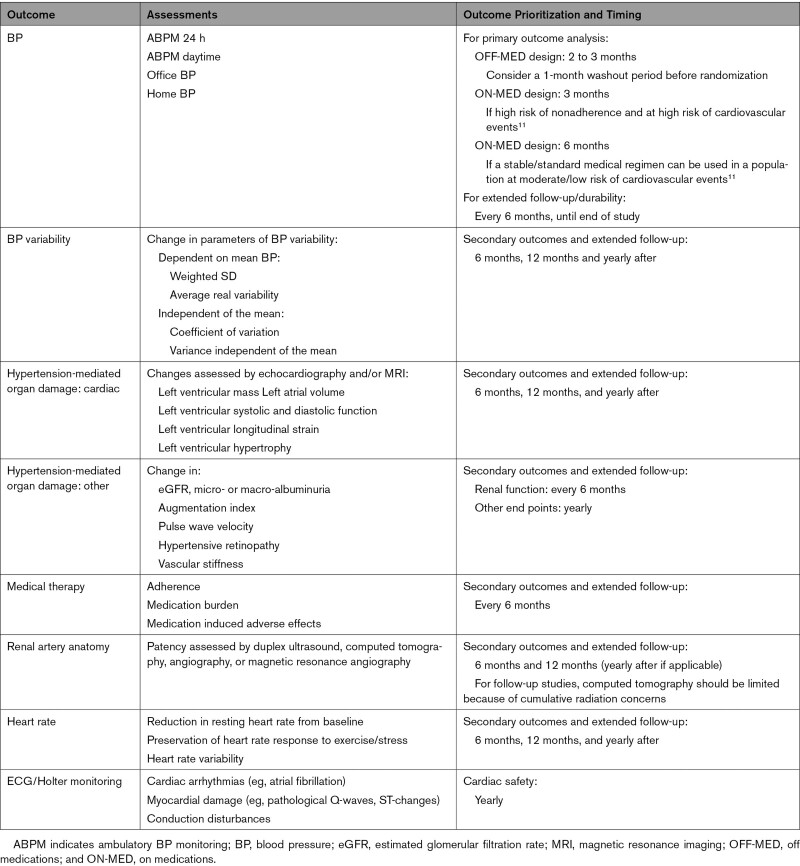
Timing for Analysis of Primary and Secondary Outcomes in Hypertension Trials With Device-Based Therapies

Available evidence supporting the use of different technologies to inhibit the sympathetic nervous system reveals significant reductions in both OBP and ABPM within the first 3 months of treatment. Of note, the risk for confounding by medication adherence issues rapidly increases within the first 6 months, supporting a rationale for primary outcomes sooner rather than later within a 6-month window. In patients who are enrolled in device-based therapy trials and prescribed antihypertensive medications using stable and standardized concomitant medication regimens, selection of BP outcomes between 2 and 6 months after intervention is reasonable. For proof-of-concept studies in medication-naive patients (including participants in whom antihypertensive medications are discontinued before enrollment, for example, after a prespecified washout period), 2 to 3 months for the timing of outcome assessment may be acceptable. A 4- to 6-week washout period was shown to be safe in relatively low-risk patients with hypertension enrolled in 3 sham-controlled randomized trials,^21,22^ consistent with safety of antihypertensive medication withdrawal reported in a meta-analysis of 66 pharmaceutical trials.^75^

For trial outcomes other than BP, later time intervals may be reasonable considering the type of event assessed; for instance, atrial fibrillation burden could be assessed (ie, Holter) at 3 months, whereas assessment of left ventricular remodeling may require 6 to 12 months.^60^ Extension of the follow-up beyond 12 months is likely to be necessary for clinical outcomes (eg, death, stroke, myocardial infarction) in adequately powered studies.^7^

### Late-Term BP Outcomes and Durability of Treatment Effect

The demonstration of a sustained reduction in BP that can be solely attributed to an interventional procedure can be challenging because of medication changes, comorbidities, and patient behavior (eg, weight loss, exercise, diet, adherence).^76^ Although BP assessment after a medication washout period at late-term follow-up (eg, 1 year) has been proposed to assess durability in randomized trials, comparisons can nevertheless be confounded by factors unrelated to medical therapy.

Aside from measurement of BP, quantification of antihypertensive medication use at baseline and during longitudinal follow-up is important to patients, providers, and the health care ecosystem. Against the background of sustained reductions in BP, the number of medications and doses are expected to remain stable, if not decline. The number of prescribed medications is 1 important factor that limits adherence. If the procedure is successful, decreased medication burden may lead to fewer side effects and improve adherence to the remaining medications. Just as with BP, however, medication burden might be confounded by successful healthy lifestyle changes such as weight loss, exercise, and introduction of low-fat and low-salt diets supporting withdrawal of medications. However, as recurrence of hypertension is commonplace, serial assessment during long-term follow-up for the need for medication reinstatement is an important consideration. For assessment of durability, measurement of BP at least 12 months after intervention and compared with baseline (and a control group, if possible) can provide an assurance of a durable treatment effect. Alternatively, time in a targeted BP range during serial follow-up may provide some insight into durability and has been predictive of cardiovascular risk.^66^

### Late-Term Safety Outcomes

Kidney function is a critical safety issue associated with both hypertension and RDN. After RDN, measurements of kidney function (ie, estimated glomerular filtration rate) are advocated at 6-month intervals for up to 3 years. A secondary consideration is the measurement of the urine albumin/creatinine ratio, because increases in micro- or macro-albuminuria are associated with an adverse kidney and cardiovascular prognosis.^77^

A more challenging issue is the assessment of renal artery anatomy after an endovascular procedure because of variability in noninvasive assessment procedures and the need to differentiate morphological changes from the natural history of atherosclerotic disease progression. Currently, duplex ultrasound, computed tomographic angiography, and magnetic resonance angiography have been applied with reasonable success in the existing device-based hypertension trials.^19–23^ With current RDN therapies, development of device-related proximal renal artery stenosis has been rarely observed.^20–22,78^ Among such cases reported in the context of both clinical trials and routine practice, the majority occur within the first year after treatment.^78^ Therefore, assessment of renal vasculature at 6 or preferably 12 months is recommended. On the imaging modality (eg, ultrasound, computed tomographic angiography, magnetic resonance angiography, catheter-based angiography), it is recommended to use the imaging procedure that is most feasible and is associated with high levels of study interpretation expertise within the particular health system. Escape of BP control or an unexplained decrease in the estimated glomerular filtration rate can be a useful clinical indicator of renal artery damage or dysfunction, prompting further study. Duplex ultrasound will detect only hemodynamically significant lesions and is not optimal for assessment of distal branch lesions. Pending the establishment of long-term safety associated with RDN, patient follow-up for at least several years is reasonable.

## Clinical Trial Target Populations

### Severity of Hypertension

Incorporating the methodological lessons learned from SYMPLICITY HTN-3,^17^ contemporary randomized RDN trials have enrolled patients with moderate but uncontrolled hypertension (eg, average OBP ~155/100 mm Hg) in the presence and absence of medical therapy.^19–23^ In contrast, the SYMPLICITY HTN-3 trial enrolled patients with severe, treatment-resistant hypertension (average OBP ~180/98 mm Hg despite >5 prescribed antihypertensive medications).^17^ On the basis of more contemporary trials, RDN may have utility as an adjunct to tailored medical therapy to improve BP control and reduce pill burden.^44^ Among contemporary randomized trials, treatment with RDN has been associated with at least similar reduction in office SBP to what has been achieved with a single antihypertensive medication.^8^ However, whereas SYMPLICITY HTN-3 was performed in truly treatment-resistant severe hypertension, several subsequent studies were performed either OFF-MED or in more moderate hypertension ON-MED scenarios of uncontrolled hypertension. Thus, robust evidence for the utility of RDN in severe resistant hypertension is lacking and needs to be explored in further randomized controlled trials considering the associated poor medication adherence. For instances of severe hypertension, RDN may achieve BP control with existing medications or potentially reduce the medication burden, but in most cases will be complementary rather than exclusionary to medications for BP control. Alternatively, preference for nonpill or reduced lifetime medication burden and associated side effects are characteristics of RDN candidates in the earlier stages of the hypertensive disease.^44^ In these settings, RDN could become a secondary alternative to starting with medications in patients with mild hypertension in the short term, but medication(s) may also be needed to control BP during long-term follow-up.

### Isolated Systolic Hypertension

Isolated systolic hypertension (ISH) has been associated with a reduced response to RDN,^79^ which, if confirmed in future trials, may be related to an important irreversible vascular component of hypertension in these patients. On the basis of this earlier observation, patients with ISH were excluded from recent trials. However, real-world registry data^80^ as well as a subanalysis of the RADIOSOUND trial (A Randomized Comparison of Ultrasound Based Versus Radiofrequency Based Catheter Ablation Techniques in Patients With Therapy Resistant Arterial Hypertension With Large Renal Arteries)^81^ demonstrated comparable effectiveness of RDN in both ISH and combined hypertension in patients <75 years old. Also, recent insights into the BP-lowering mechanism of RDN propose reduced vascular stiffness^82^ as well as normalization of high stroke volume^83^ as additional contributors to improved BP control after RDN. These modes of action could equally apply in patients with ISH. The presumption of a reduced BP-lowering effect of RDN in patients with ISH may have been overestimated and might also not hold true for newer denervation technologies and techniques.

### White-Coat Hypertension and Other Hyperadrenergic Hypertensive Conditions

Primary hypertension is characterized by an increase in sympathetic activity combined with other neuroendocrine, kidney, vascular, central, genetic, and environmental mechanisms. However, in specific hypertensive conditions, sympathetic activation appears to be more pronounced and represents a hallmark of the high BP state. This is the case for white-coat hypertension, in which increased OBP with normal 24-hour ABP or home BP has been associated with a marked adrenergic activity as assessed by microneurographic recording of sympathetic nerve traffic.^84,85^ The influence of RDN on these various BP phenotypes is not well characterized and represents an opportunity for dedicated study.

### Hypertension-Mediated Organ Damage and Patients at High Cardiovascular Risk

Data from observational studies suggest that RDN may be associated with favorable changes in diastolic function indices and left ventricular mass.^60,61^ Kidney function is neutrally or beneficially influenced by RDN, whereas for arterial structure and function, the data are scarce.^86^ Given the close link of left ventricular mass changes with improved outcomes in hypertension,^11^ patients with left ventricular hypertrophy and unfavorable remodeling could be a target RDN population. High cardiovascular risk settings such as coronary artery disease, diabetes, and patients with chronic kidney disease with uncontrolled hypertension^11^ would be expected to have the greatest benefit from BP control by RDN.^44^ In addition, hypertension with other comorbidities such as heart failure and arrhythmia burden (ie, atrial fibrillation) are particularly relevant populations for future studies.^44,80^ Because difficult-to-control hypertension is related to cerebrovascular events (of high concern for patients) and BP control is linked to better outcomes,^7^ improvement of cognitive decline and stroke risk could be core outcomes for inclusion in RDN trials.

### Indications Beyond Hypertension

Because RDN interrupts both renal efferent and afferent nerve activity, effects on reducing the sympathetic signature within the body may have pluripotent effects beyond BP reduction (Figure S1). Although hypertension has remained the focus of major clinical trials for RDN, several observational and small randomized studies have explored its potential in disease states other than hypertension, including arrhythmias,^67,87^ glucose metabolism,^88^ obstructive sleep apnea,^89^ chronic kidney disease,^90^ and heart failure.^91^ Although such studies remain preliminary, HARC advocates similar trial design, conduct, and attention to safety as with hypertension trials, although primary outcomes may differ.

## Statistical Considerations

Randomized controlled trials of device-based therapies for hypertension have consistently used change in BP measurement (generally 24-hour or daytime systolic ABP change from baseline to 2–6 months) as the primary outcome. ANCOVA adjusted on baseline BP is preferable to a simple paired *t* test to test the primary hypothesis (as reported in the next generation trials),^92^ and standard statistics are used to report binary variables, multilevel categorical variables, and time-to-event clinical outcomes. Four considerations are essential during the design phase. First, given the general use of a sham control in current trials, the assumed difference in treatment outcomes and the associated sample size considerations must account for an expected reduction in the sham group. Second, the intention-to-treat population should be considered as the primary analysis cohort; however, there are legitimate reasons to analyze a per-protocol population in addition (eg, excluding untreated patients, those with major protocol violations, those missing ABPM, or those fulfilling escape criteria requiring change in medications), and the as-treated cohort. Third, given uncertainty about the effects of novel device-based therapies for hypertension, Bayesian adaptive designs have been proposed as a more efficient method that avoids unnecessary enrollment of patients.^22^ This approach allows for prespecified interim analyses with predetermined stopping rules for effectiveness or futility.^22^ However, it increases statistical complexity and reduces sample size, but a larger sample might be important for the detection of infrequent safety outcomes. Fourth, in an effort to incorporate clinical outcomes in the primary outcome of device-based therapies for hypertension trials, generalized pairwise comparison methods can be used to hierarchically assess components of a composite. Offering advantages beyond traditional time-to-event analyses, generalized pairwise comparison methods, which include the Finkelstein-Schoenfeld and win-ratio methods, offer the advantages of prioritizing most severe outcomes, incorporating recurrent events, and combining time-to-event with quantitative outcomes.^93^ In addition, such methods allow the combination of BP outcomes with patient-centered outcomes, such as medication burden.^94^

## Conclusions

The advancement of device-based approaches to hypertension has been motivated by

clinical implications associated with a persistently high prevalence of both uncontrolled BP and medication nonadherence. The expansion of device technologies and emerging clinical trials imparts the need for consistency in trial design, conduct, and definitions of clinical study elements to allow for trial comparability and data poolability. The HARC program represented an integration of evolving evidence and consensus opinion among leading experts in cardiovascular medicine and hypertension research with regulatory perspective on clinical trial design and methodology. From this program, clinical design principles and outcomes definitions for studies aimed at evaluating device-based hypertension therapies were established, and standardization of BP assessment, effectiveness measures beyond BP, and safety outcomes have been proposed.

## Article Information

HARC Steering Committee: David E. Kandzari, MD; Felix Mahfoud, MD; Michael A. Weber, MD; Konstantinos Tsioufis, MD; Donald Cutlip, MD; and Ernest Spitzer, MD.

### Acknowledgments

The authors are grateful to Hans Jonker at Cardialysis for preparation of the figures.

### Sources of Funding

HARC was funded through grants from Medtronic, ReCor Medical, Boston Scientific, BackBeat Medical, Ablative Solutions, Vascular Dynamics, and Metavention.

### Disclosures

Dr Kandzari reports institutional research/grant support from Ablative Solutions and Medtronic; and personal consulting honoraria from Medtronic. Dr Mahfoud receives research support from the Deutsche Gesellschaft für Kardiologie and Deutsche Forschungsgemeinschaft and has received scientific support and speaker honoraria from Bayer, Boehringer Ingelheim, Medtronic, and ReCor Medical. Dr Weber reports consulting honoraria from Urovant, Johnson & Johnson, Ablative Solutions, Regeneron, Medtronic, Omron, and ReCor Medical. Dr Townsend receives consultant honoraria from Medtronic and Regeneron and royalties from UpToDate and is a data and safety monitoring member at AXIO. Dr Parati declares speaking honoraria from Omron Healthcare, Servier, and Novartis. Dr Fisher receives consultant and research funding from Recor Medical and consulting honoraria from Medtronic and Aktiia. Dr Lobo receives consulting honoraria from Medtronic, ReCor Medical, Ablative Solutions, CVRx, Rox Medical, and Vascular Dynamics and reports educational grants from Medtronic and ReCor Medical. Dr Bloch reports consulting honoraria from Recor and Medtronic. Dr Böhm reports research support from the Deutsche Forschungsgemeinschaft and reports personal fees from Abbott, Amgen, AstraZeneca, Bayer, Boehringer Ingelheim, Cytokinetics, Daiichii-Sankyo, Medtronic, Novartis, ReCor, Servier, and Vifor. Dr Azizi receives research grants from the French Ministry of Health, Quantum Genomics, and the European Horizon 2020 program; grant support and nonfinancial support from ReCor Medical and Idorsia; and personal fees from CVRx, AstraZeneca, Alnylam Pharmaceutical, and Poxel Pharma. Dr Schlaich receives research supported from the NHMRC Research Fellowship and has received consulting fees or travel and research support from Medtronic, Abbott, Otsuka, Novartis, Servier, Pfizer, and Boehringer Ingelheim. Dr Kirtane reports institutional funding to Columbia University or the Cardiovascular Research Foundation from Medtronic, Boston Scientific, Abbott Vascular, Amgen, CSI, Siemens, Philips, ReCor Medical, Neurotronic, and Biotronik. In addition to research grants, institutional funding includes fees paid to Columbia University or Cardiovascular Research Foundation for consulting or speaking engagements in which Dr Kirtane controlled the content. Dr Kirtane also reports consulting for IMDS; and travel expenses/meals from Medtronic, Boston Scientific, Abbott Vascular, Abiomed, CSI, Siemens, Philips, ReCor Medical, Chiesi, OpSens, Zoll, and Regeneron. Dr Daemen receives institutional grant/research support from AstraZeneca, Abbott Vascular, Boston Scientific, ACIST Medical, Medtronic, Pie Medical, and ReCor Medical. Dr Pathak declares consulting honoraria from Medtronic, ReCor Medical, Ablative Solutions, and CVRx and educational and research grants from Medtronic, ReCor Medical, and Ablative Solutions. Dr Ukena reports consulting and lecture honoraria from Bayer, Boehringer Ingelheim, Medtronic, and Pfizer. Dr Lurz declares institutional grants from ReCor Medical. Dr Grassi declares lecture honoraria from Medtronic and Merck. Dr Finn receives research grants from ReCor Medical and Medtronic through CVPath, and honoraria from Medtronic. Dr Morice declares being the chief executive officer and shareholder of CERC. Dr Jüni serves as an unpaid steering committee member of trials funded by Abbott Vascular, AstraZeneca, Biotronik, Biosensors, St Jude Medical, Terumo, and The Medicines Company; receives institutional research grants from Appili Therapeutics, AstraZeneca, Biotronik, Biosensors International, Eli Lilly, and The Medicines Company; and receives institutional honoraria for participation in advisory boards or consulting from Amgen, Ava, and Fresenius, but has not received personal payments by any pharmaceutical company or device manufacturer. Dr Stone has received speaker or other honoraria from Terumo, Cook, and Infraredx; has served as a consultant to Valfix, TherOx, Robocath, HeartFlow, Ablative Solutions, Vectorious, Miracor, Neovasc, Abiomed, Ancora, Elucid Bio, Occlutech, CorFlow, Reva, MAIA Pharmaceuticals, Vascular Dynamics, Shockwave, V-Wave, Cardiomech, and Gore; and has equity/options from Ancora, Cagent, Applied Therapeutics, the Biostar family of funds, SpectraWave, Orchestra Biomed, Aria, Cardiac Success, Valfix, and the MedFocus family of funds. The other authors report no conflicts.

### Supplemental Material

HARC Participants

Tables S1–S9

Figure S1

## Supplementary Material
