# Supplementary material for: Clinical Trial Design Principles and Outcomes Definitions for Device-Based Therapies for Hypertension: A Consensus Document From the Hypertension Academic Research Consortium
Source: Circulation. 2022 Mar 15;145(11):847–63. doi: 10.1161/CIRCULATIONAHA.121.057687 (PMC8912966; doi:10.1161/CIRCULATIONAHA.121.057687)

SUPPLEMENTARY APPENDIX

# Clinical Trial Design Principles and Endpoint Definitions for Device-based Therapies for Hypertension: A Consensus Document from the Hypertension Academic Research Consortium

A Consensus Document from the Hypertension Academic Research Consortium (HARC)

Steering Committee: David E. Kandzari, MD; Felix Mahfoud, MD; Michael A. Weber, MD; Konstantinos Tsioufis, MD; Donald Cutlip, MD; Ernest Spitzer, MD.

Authors:

David E. Kandzari, MD[1]; Felix Mahfoud, MD[2,3]; Michael A. Weber, MD[4]; Raymond Townsend, MD[5]; Gianfranco Parati, MD[6,7]; Naomi D. L. Fisher, MD[8]; Melvin D. Lobo, MD[9]; Michael Bloch, MD[10,11]; Michael Böhm, MD[2]; Andrew S.P. Sharp, MD[12]; Roland E. Schmieder, MD[13]; Michel Azizi, MD[14]; Markus P. Schlaich, MD[15]; Vasilios Papademetriou, MD[16]; Ajay J. Kirtane, MD, SM[17,18]; Joost Daemen, MD, PhD[19,20]; Atul Pathak, MD, PhD[21]; Christian Ukena, MD[2]; Philipp Lurz, MD, PhD[22]; Guido Grassi, MD[23]; Martin Myers, MD[24]; Aloke V. Finn, MD[25]; Marie-Claude Morice, MD[26]; Roxana Mehran, MD[18,27]; Peter Jüni, MD[28]; Gregg W. Stone, MD[18,27]; Mitchel Krucoff, MD[29]; Paul K. Whelton, MD[30]; Konstantinos Tsioufis, MD[31]; Donald E. Cutlip, MD[32,33]; Ernest Spitzer, MD[19,20].

**Author Affiliations:**

[1]Piedmont Heart Institute, Atlanta, GA, USA;

[2]Klinik für Innere Medizin III, Kardiologie, Angiologie und Internistische Intensivmedizin, Universitätsklinikum des Saarlandes, Saarland University, Homburg, Germany;

[3]Institute for Medical Engineering and Science, Massachusetts Institute of Technology, Cambridge, MA, USA;

[4]State University of New York, Downstate Medical College, New York, New York, USA

[5]University of Pennsylvania, Perelman School of Medicine, Philadelphia, PA, USA

**SUPPLEMENTARY APPENDIX**

[6]Department of Medicine and Surgery, University of Milano-Bicocca, Milan, Italy

[7]Istituto Auxologico Italiano IRCCS, Ospedale San Luca, Milan, Italy

[8]Brigham and Women's Hospital, Boston, Massachusetts, USA

[9]Barts NIHR Biomedical Research Centre, William Harvey Research Institute, Queen Mary University of London, London, United Kingdom

[10]University of Nevada/Reno School of Medicine, Reno, Nevada, USA

[11]Renown Institute for Heart and Vascular Health, Reno, Nevada, USA

[12]University Hospital of Wales, Cardiff and University of Exeter, Exeter, United Kingdom

[13]Department of Nephrology and Hypertension, University Hospital Erlangen, Friedrich Alexander University Erlangen/Nürnberg, Germany

[14]University of Paris, INSERM, CIC1418; AP-HP Hypertension Department and DMU CARTE, Georges Pompidou European Hospital, Paris, France

[15]Dobney Hypertension Centre, School of Medicine - Royal Perth Hospital Unit and Research Foundation, University of Western Australia, Perth, WA, Australia.

[16]Department of Veterans Affairs and Georgetown University Medical Centers, Washington DC, USA

[17]Columbia University Irving Medical Center/New York-Presbyterian Hospital, New York, USA

[18]Cardiovascular Research Foundation, New York, USA

[19]Cardialysis, Rotterdam, The Netherlands

[20]Thoraxcenter, Department of Cardiology, Erasmus University Medical Center, Rotterdam, The Netherlands

[21]Department of Cardiovasculaire Medicine, ESH Hypertension Excellence Center, Princess Grace Hospital, Monaco; and UMR UT3 CNRS 5288, Toulouse, France

[22]Heart Center Leipzig at University of Leipzig, Leipzig, Germany

[23]Clinica Medica University Milano-Bicocca, Milan, Italy

[24]Division of Cardiology, Sunnybrook Health Sciences Centre, University of Toronto, Canada

[25]CVPath Institute, Gaithersburg, Maryland, USA

[26]CERC, Paris, France

[27]Mount Sinai Hospital, New York, New York, USA

[28]Li Ka Shing Knowledge Institute of St. Michael's Hospital, Toronto, Ontario, Canada

[29]Duke Clinical Research Institute, Durham, North Carolina, USA

[30]Departments of Epidemiology and Medicine, Tulane University Health Sciences Center, New Orleans, Louisiana, USA.

[31] 1st Department of Cardiology, National and Kapodistrian University of Athens, Hippocratio Hospital, Greece

[32]Baim Institute for Clinical Research, Boston, Massachusetts, USA

[33]Beth Israel Deaconess Medical Center, Boston, Massachusetts, USA

**Address for correspondence:**

David E. Kandzari, MD, Piedmont Heart Institute, Suite 2065, 95 Collier Road, Atlanta, GA 30309; Tel: +404 605 5108; Fax: +404 720 0911; Email: david.kandzari@piedmont.org; @Kandzari

**SUPPLEMENTARY APPENDIX**

**List of participants**

HARC Scientific Committee:

1. David Kandzari (US)
2. Felix Mahfoud (DE)
3. Michael Weber (US)
4. Konstantinos Tsioufis (GR)
5. Don E. Cutlip (BICR, US)
6. Ernest Spitzer (Cardialysis, NL)

HARC Faculty:

1. Ajay J. Kirtane (US)
2. Aloke V. Finn (US)
3. Andrew S. P. Sharp (UK)
4. Atul Pathak (MC)
5. Christian Ukena (DE)
6. Gianfranco Parati (IT)
7. Gregg W. Stone (US)
8. Guido Grassi (IT)
9. Joost Daemen (NL)
10. Markus P. Schlaich (AU)
11. Martin Myers (US)
12. Melvin D. Lobo (UK)
13. Michael Bloch (US)
14. Michael Böhm (DE)
15. Michael A. Weber (US)
16. Michel Azizi (FR)
17. Naomi D. L. Fisher (US)
18. Paul K. Whelton (US)
19. Peter Jüni (CA)
20. Philipp Lurz (DE)

21. Raymond Townsend (US)
22. Roland E. Schmieder (DE)
23. Vasilios Papademetriou (US)

Regulatory representatives:

1. Andrew Farb (US FDA)
2. Bram Zuckerman (US FDA)
3. Brian Pullin (US FDA)
4. Finn Donaldson (US FDA)
5. Hiren Mistry (US FDA)
6. Robert Lee (US FDA)
7. Pedro Eerdmans (NL DEKRA)

Academic Research Consortium:

1. Andrew Farb (US FDA)
2. Donald E. Cutlip (US)
3. Ernest Spitzer (NL)
4. Marie-Claude Morice (FR)
5. Mitchell Krucoff (US)
6. Roxana Mehran (US)

Grant-givers:

1. Medtronic
2. ReCor Medical
3. Boston Scientific
4. BackBeat Medical
5. Ablative Solutions
6. Vascular Dynamics
7. Metavention

HARC Industry Participants:

1. Denise Jones (Medtronic)
2. Doug Hettrick (Medtronic)
3. Gabriel Lazarus (Medtronic)
4. Jason Fontana (Medtronic)
5. Julie Trudel (Medtronic)
6. Manuela Negoita (Medtronic)
7. Sandeep Brar (Medtronic)
8. Sidney Cohen (Medtronic)
9. Vanessa DeBruin (Medtronic)

10. Andrew Weiss (ReCor Medical)
11. Helen Reeve-Stoffer (ReCor Medical)
12. Neil Barman (ReCor Medical)
13. Kazumichi Kobayashi (Otsuka/ReCor Medical)
14. Yusuke Kogata (Otsuka/ReCor Medical)
15. Nicholas Mahowald (Boston Scientific)
16. Daniel Burkhoff (BackBeat Medical)
17. Yuval Mika (BackBeat Medical)
18. Nicole Haratani (Ablative Solutions)
19. John McIntyre (Vascular Dynamics)
20. Todd Berg (Metavention)

**Supplementary Table 1.** Methods to detect adherence to antihypertensive medication.

| Method | Description | Advantage | Disadvantage |
|---|---|---|---|
| **Direct methods** | | | |
| Drug assay (first choice) | Measurement of drug or metabolite levels in plasma/urine | Quantitative<br>Objective<br>Reliable<br>May be feasible in oral fluids or dried blood samples | Costly<br>Not routinely available<br>Not routinely available<br>In trials with multiple BP drugs, urine AND blood sampling may be needed<br>False positives and negatives are possible |
| Directly observed therapy | Medication administered under supervision of clinical staff | Quantitative<br>Objective | Costly<br>Resource intensive<br>Risk of severe hypotension<br>Relevant only to the days of observation |

| | | | |
|---|---|---|---|
| Digital Medicine | Biosafe sensor incorporated in pill is activated in stomach and sends signal to patch worn by patient | Quantitative<br>Objective<br>Reliable | Costly<br>Not routinely available |
| **Indirect methods** | | | |
| Interview | Patient interview by doctor/nurse/allied health professional | Simple<br>Inexpensive<br>Easily available | Qualitative<br>Unreliable<br>Non-objective<br>Time consuming |
| Diary/self-report patient questionnaire | Questionnaire provides structure to patient diary and self-reports | Simple<br>Inexpensive<br>Easily available | Qualitative<br>Unreliable<br>Non-objective<br>Time consuming |
| Pill count | Patient returns pill box to medical facility | Quantitative<br>Simple<br>Inexpensive<br>Easily available | Poor reliability<br>Non-objective |

| | | | |
|---|---|---|---|
| Refill data | Calculation of percentage of days covered by prescription enables approximation of adherence/persistence | Quantitative | Poor reliability<br>Poor objectivity<br>Costly<br>Not routinely available |
| Assessment of response or physiological markers | Evaluation of BP response (e.g., telemedicine) or measurement of markers (e.g., heart rate or biochemical parameters) | Quantitative<br>Moderate objectivity<br>Reliable | Costly<br>Resource intensive<br>Poor reliability<br>Poor objectivity |
| Electronic drug monitoring systems | Electronic pillbox is activated when opened and drug removed | Quantitative<br>Objective<br>Reliable | Costly<br>Not routinely available |

BP indicates blood pressure.

**Supplementary Table 2.** An example of structure drug titration during renal denervation trials.

| Step (Target systolic BP < 140 mmHg) | Drug | Treatment Score |
|---|---|---|
| 0 (not needed) | None | 0 |
| 1 (if needed) | Calcium channel blocker, mid-dose | 1 |
| 2 (if needed) | ACE inhibitor or ARB, full-dose | 2 |
| 3 (if needed) | Hydrochlorothiazide 12.5 mg | 3 |
| 4 (if needed) | Hydrochlorothiazide 25 mg | 4 |
| 5 (if needed) | Calcium channel blocker, full-dose | 5 |
| 6 (if needed) | Spironolactone or BB or clonidine | 6 |
| 7 (if needed) | Spironolactone or BB or clonidine | 7 |
| 8 (if needed) | Spironolactone or BB or clonidine | 8 |

ACE indicates angiotensin-converting enzyme; ARB, angiotensin-receptor blocker; BB, beta-blocker; BP, blood pressure.

Consider 2 to 3 weeks between steps. If target is reached, no further steps even if BP fluctuates above target. For steps 6 through 8, choice of drug and dose at investigator's discretion. If initial systolic BP ≥

160 mmHg, steps 1 and 2 can be combined. Fixed-combination drug products can be used to decrease pill burden. In protocols where patients already receive drugs, step sequence will begin between steps 2 and 6 depending on number of drugs in the ongoing regimen. Adapted from Weber et al. J Clin Hypertens. 2015;17:743-750.

**Supplementary Table 3.** Selected advantages and disadvantages of home blood pressure measurements and ambulatory blood pressure monitoring in device-based therapies for hypertension trials.

| **Ambulatory Blood Pressure Monitoring** | **Home Blood Pressure** |
|---|---|
| **Advantages** | |
| Identification of white-coat and masked hypertension | Identification of white-coat and masked hypertension |
| Diagnosis of true resistant hypertension, excluding white coat | Diagnosis of true resistant hypertension, excluding white coat |
| Assessment of night-time BP | Repeated measurements in a standardized home setting |
| High reproducibility of average ambulatory BP values | High reproducibility of average HBP values |
| Limited placebo effect; no observer bias | Limited placebo effect; no observer bias |
| Real-life settings | Assessment of long term RDN effects |
| Assessment of RDN effects on 24h BP patterns* | Assessment of RDN effects on day-to-day BP variability and visit-to-visit variability |
| Assessment of daytime and nighttime variability | |
| Strong evidence of prognostic value for 24h, day and night BP, whose measures thus represent suitable endpoints for RDN | Increasing evidence for prognostic value of average HBP, whose measure thus represents a suitable endpoint for RDN |
| **Disadvantages** | |
| Can be uncomfortable, burdensome | Only BP at rest and at home is available |
| Can disrupt sleep | Potential for measurement and reporting errors** |

| | |
|---|---|
| Limited reproducibility of individual readings | No nocturnal readings (most devices) |
| Non-standardized behavioral conditions, open to noise interference | Need for patient compliance with one week measurement schedule, 2 BP measurements 1 minute apart after 5 minutes rest in the morning and in the evening |

* Nocturnal dipping, morning surge, short-term BP variability.

** Need for tele-monitoring facilities and/or device memory function.

BP indicates blood pressure; RDN, renal denervation.

**Supplementary Table 4.** Comparison of main features of home blood pressure monitoring, office blood pressure measurement and ambulatory blood pressure monitoring in hypertension trials.

| **Feature** | **HBPM** | **OBPM** | **ABPM** |
|---|---|---|---|
| Standardized assessment of baseline and follow-up BP in a clinic setting, especially for unattended BP measurements | - | ++ | - |
| Assessment of baseline and follow-up daytime BP | ++ | NA | ++ |
| Assessment of baseline and Follow-up night-time BP level and dipping pattern | +* | - | +++ |
| Assessment of baseline and follow-up morning surge BP | +* | - | ++ |
| Assessment of baseline and follow-up morning hypertension | ++ | +/- | +++ |
| Assessment of baseline and follow-up 24 h BP | - | - | +++ |
| Number of BP measurements obtainable | ++ | + | +++ |
| Placebo effect | - | + | - |
| Observer bias elimination | +++**,*** | +** | +++ |
| Increase in study power and reduction in sample size | +++ | + | +++ |
| Subjects selection | +++ | + | +++ |

| | | | |
|---|---|---|---|
| Diagnosis of true resistant hypertension | ++ | + | +++ |
| Detection of white-coat hypertension | +++ | + | +++ |
| Detection of masked hypertension | ++ | + | +++ |
| Assessment of short-term BPV | + (Morning-evening) | + (Within visit) | +++ (24h) |
| Assessment of mid-term BPV | +++ (day by day) | + | - |
| Assessment of long-term BPV | ++ (before visit -to-before visit/ seasonal) | +++ (Visit-to-visit/ seasonal) | + (Seasonal) |
| Association with cardiovascular events risk | +++ | + | +++ |
| Assessment of duration of drug/device BP effect | + | +/- | ++ |
| Repeated monitoring in longitudinal trials | +++ | ++ | + |
| Relation with treatment induced changes in HMOD | ++ | + | +++ |
| Reproducibility in patient cohorts | ++ | - | ++ |

| | | | |
|---|---|---|---|
| Availability | ++ | ++ | - |

* Specific devices; ** Automated devices; *** Tele-monitoring.

ABPM indicates ambulatory BP monitoring; BP, blood pressure; BPV, blood pressure variability; HBPM, home blood pressure monitoring; HMOD, hypertension-mediated organ damage; OBPM, office BP measurement.

**Supplementary Table 5.** Death cause classification according to the Standardized Data Collection for Cardiovascular Trials Initiative and the US Food and Drug Administration 2017 definition.

| |
|---|
| **Cardiovascular** |
| Acute myocardial infarction |
| Sudden cardiac death |
| Heart Failure |
| Stroke |
| Cardiovascular procedures |
| Cardiovascular hemorrhage |
| Other |
| **Non-cardiovascular** |
| Pulmonary |
| Renal |
| Gastrointestinal |
| Hepatobiliary |
| Pancreatic |
| Infection (including sepsis) |

| | |
|---|---|
| | Inflammatory (e.g. systemic inflammatory response syndrome) / Immune (including autoimmune) (may include anaphylaxis from environmental, e.g. food allergies) |
| | Hemorrhage that is neither CV bleeding or a stroke |
| | Non-cardiovascular procedure or surgery |
| | Trauma (includes homicide) |
| | Suicide |
| | Non-prescription drug reaction or overdose |
| | Prescription drug reaction or overdose (may include anaphylaxis) |
| | Neurological (non-CV) (excludes CV death from ischemic stroke, hemorrhagic stroke, or undetermined cause of stroke or CV hemorrhage of central nervous system) |
| | Malignancy |
| | Other |
| **Undetermined** | |

**Supplementary Table 6.** HARC definition for vascular access site and access-related complications*.

| |
|---|
| **Major vascular complications** |
| Access site or access-related vascular injury – from the puncture site up to the renal arteries (dissection, stenosis, perforation, rupture, arterio-venous fistula, pseudoaneurysm, significant hematoma, irreversible nerve injury, compartment syndrome, percutaneous closure device failure) leading to death, life-threatening or major bleeding, visceral ischemia, or neurological impairment |
| Distal embolization from a vascular source requiring surgery or irreversible end-organ damage |
| Artery dissection or perforation requiring an unplanned endovascular or surgical intervention |
| Any new ipsilateral lower extremity ischemia documented by patient symptoms, physical exam, and/or decreased or absent blood flow on lower extremity angiogram |
| Surgery for access site-related nerve injury |
| Permanent access site-related nerve injury |
| **Minor vascular complications** |
| Access site or access-related vascular injury (dissection, stenosis, perforation, rupture, arterio-venous fistula, pseudoaneurysms, hematomas, percutaneous closure device failure) not leading to death, life-threatening or major bleeding, visceral ischemia, or neurological impairment |
| Distal embolization treated with embolectomy and/or thrombectomy and not resulting in amputation or irreversible end-organ damage |
| Any unplanned endovascular stenting or unplanned surgical intervention not meeting the criteria for a major vascular complication |

| |
|---|
| Vascular repair or the need for vascular repair (via surgery, ultrasound-guided compression, transcatheter embolization, or stent-graft) |
| Failure of a closure device to achieve hemostasis at the arteriotomy site leading to alternative treatment (other than manual compression or adjunctive endovascular ballooning) |

*Modified from VARC-2

**Supplementary Table 7.** HARC definition for bleeding events.

| **HARC Primary Bleeding Scale (modified from VARC-2)** |
|---|
| **Life-threatening or disabling bleeding** |
| Fatal bleeding (BARC type 5) OR |
| Bleeding in a critical organ, such as intracranial, intraspinal, intraocular, or intramuscular with compartment syndrome (BARC type 3b and 3c) OR |
| Bleeding causing hypovolemic shock or severe hypotension requiring vasopressors or surgery (BARC type 3b) OR |
| Overt source of bleeding with drop in hemoglobin ≥5 g/dl or whole blood or packed red blood cells (RBCs) transfusion ≥4 units (BARC type 3b) |
| **Major bleeding (BARC type 3a)** |
| Overt bleeding either associated with a drop in the hemoglobin level of at least 3.0 g/dl or requiring transfusion of two or three units of whole blood/RBC, or causing hospitalization or permanent injury, or requiring surgery AND does not meet criteria of life-threatening or disabling bleeding |
| **Minor bleeding (BARC type 2 or 3a, depending on the severity)** |
| Any bleeding worthy of clinical mention (e.g., access site hematoma) that does not qualify as life-threatening, disabling, or major |
| **HARC Secondary Bleeding Scale (modified from BARC)** |
| **Type 0:** no bleeding |
| **Type 1**: bleeding that is not actionable and does not cause the patient to seek unscheduled performance of studies, hospitalization, or treatment by a healthcare |

professional; may include episodes leading to self-discontinuation of medical therapy by the patient without consulting a healthcare professional

**Type 2:** any overt, actionable sign of hemorrhage (eg, more bleeding than would be expected for a clinical circumstance, including bleeding found by imaging alone) that does not fit the criteria for type 3, 4, or 5 but does meet at least one of the following criteria: (1) requiring nonsurgical, medical intervention by a healthcare professional, (2) leading to hospitalization or increased level of care, or (3) prompting evaluation

**Type 3**

**Type 3a**

Overt bleeding plus hemoglobin drop of 3 to 5 g/dL* (provided hemoglobin drop is related to bleed)

Any transfusion with overt bleeding

**Type 3b**

Overt bleeding plus hemoglobin drop 5 g/dL* (provided hemoglobin drop is related to bleed)

Cardiac tamponade

Bleeding requiring surgical intervention for control (excluding dental/nasal/skin/hemorrhoid)

Bleeding requiring intravenous vasoactive agents

**Type 3c**

Intracranial hemorrhage (does not include microbleeds or hemorrhagic transformation, does include intraspinal).

Subcategories confirmed by autopsy or imaging or lumbar puncture

Intraocular bleed compromising vision

**Type 4 (periprocedural):**

Perioperative intracranial bleeding within 48 h

Reoperation after closure of incision site for the purpose of controlling bleeding

Transfusion of 5 U whole blood or packed RBSs within a 48-h period of the procedure

**Type 5:** fatal bleeding

**Type 5a**

Probable fatal bleeding; no autopsy or imaging confirmation but clinically suspicious

**Type 5b**

Definite fatal bleeding; overt bleeding or autopsy or imaging confirmation

---

*Corrected for transfusion (1 U packed red blood cells or 1 U whole blood 1 g/dL hemoglobin).

**Supplementary Table 8.** HARC definition for acute kidney injury*.

| |
|---|
| **Stage 1** |
| Increase in serum creatinine to 150–199% (1.5–1.99 × increase compared with baseline) OR increase of ≥0.3 mg/dl (≥26.4 mmol/l) OR |
| Urine output <0.5 ml/kg/h for >6 but <12 h |
| **Stage 2** |
| Increase in serum creatinine to 200–299% (2.0–2.99 × increase compared with baseline) OR |
| Urine output <0.5 ml/kg/h for >12 but <24 h |
| **Stage 3** |
| Increase in serum creatinine to ≥300% (>3 × increase compared with baseline) OR |
| Serum creatinine of ≥4.0 mg/dl (≥354 mmol/l) with an acute increase of at least 0.5 mg/dl (44 mmol/l) OR |
| Urine output <0.3 ml/kg/h for ≥24 h OR |
| Anuria for ≥12 h |
| **Stage 4** |
| Need for renal replacement therapy |

*Modified from VARC-2

**Supplementary Table 9.** Recommendations for other endpoint targets in device-based therapies for hypertension trials.

| Endpoint parameter | Proposed target | Comments | Recommendations |
|---|---|---|---|
| **Visit-to-visit BP Variability** | Change in parameters of BP variability:<br>Dependent on mean BP:<br>Weighted standard deviation<br>Average real variability<br>Independent of the mean:<br>Coefficient of variation<br>Variance independent of the mean | Insufficient evidence to advise targets.<br>RDN reduces BP variability independent of the BP, and may also be a predictor of response to RDN | Assessment of RDN-induced change in BP variability is recommended in future studies of RDN. Immediate, mid-term and long-term BP variability should be differentiated. |
| **Hypertension-Mediated Organ Damage** | Change in:<br>eGFR, micro- or macro-albuminuria<br>Left ventricular mass (indexed)<br>Left ventricular systolic/ diastolic function<br>Left atrial volume (indexed)<br>Augmentation index<br>Pulse wave velocity<br>Endothelial function<br>Hypertensive retinopathy<br>Small vessel cerebrovascular disease burden<br>Carotid artery intima-medial thickness | Insufficient evidence to advise targets | Assessment of RDN-induced regression of hypertension-mediated organ damage in appropriately designed and blinded prospective RCTs is recommended as a useful surrogate endpoint in the absence of hard clinical outcome data. |

| | | | |
|---|---|---|---|
| **Heart Rate** | Reduction in resting HR from baseline | Higher resting HR may inform patient selection for RDN; data are insufficient to suggest appropriate targets for HR reduction from baseline | Assessment of RDN-induced change in resting HR is recommended in future studies of RDN |
| | Preservation of HR response to exercise/stress | HR following RDN was not blunted during exercise, indicating that RDN-induced sympatho-modulation did not adversely affect cardiac output during exercise or stress | |

BP indicates blood pressure; HR, heart rate; RDN, renal denervation.

**Supplementary Figure.** Neural control of central sympathetic activity and potential consequences

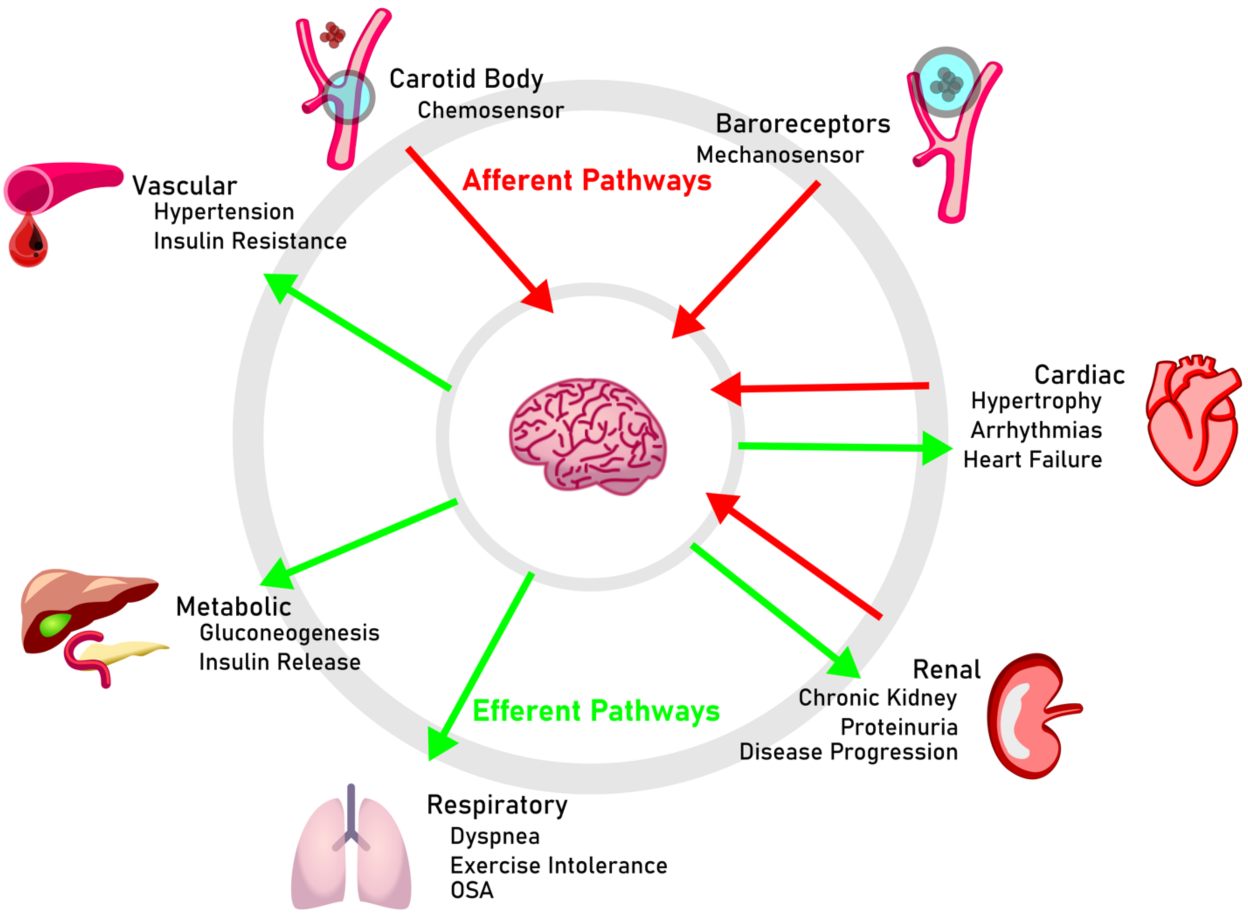

Supplement: Supplementary file 1 [file cir-145-847-s001.pdf]
